# Atypical Extramedullary Acute Myeloid Leukemia in Infancy: Bilateral Testicular Swelling and Scalp Lesions at Diagnosis

**DOI:** 10.1155/crh/2024335

**Published:** 2026-10-12

**Authors:** Ruba Alhazaimeh, Mais Jazazi, Ayman Alhwayan, Sulieman Jaafreh, Hazem Haddad, Adnan Bwaneh, Tasneem Malahmeh, Maher Khader

**Affiliations:** ^1^ Department of Pediatric Hematology-Oncology Royal Medical Services–Queen Rania Children’s Hospital Amman Jordan; ^2^ Department of Pediatric Radiology Royal Medical Services–Queen Rania Children’s Hospital Amman Jordan; ^3^ Department of Pediatric Surgery Royal Medical Services–Queen Rania Children’s Hospital Amman Jordan; ^4^ Department of Pathology and Laboratory Medicine Royal Medical Services–Princess Iman Center for Research and Laboratory Amman Jordan lhsc.on.ca

**Keywords:** acute myeloid leukemia, acute myelomonocytic leukemia, case report, myeloid sarcoma, testicular neoplasms

## Abstract

Acute myeloid leukemia (AML) is rare in infancy, with monocytic subtypes such as acute myelomonocytic leukemia often demonstrating extramedullary involvement. This report describes a 10‐month‐old male infant presenting with firm bilateral testicular enlargement and multiple parietal scalp nodules, accompanied by leukocytosis and circulating blasts in peripheral blood. Bone marrow examination confirmed French–American–British (FAB) M4 AML. Scrotal ultrasound demonstrated heterogeneous masses, and computed tomography identified scalp chloromas without bone erosion. The patient received AML chemotherapy, resulting in resolution of extramedullary lesions and achievement of remission. This case underscores the importance of early hematologic evaluation in infants with bilateral testicular masses to prevent misdiagnosis. It illustrates the rare occurrence of concurrent testicular and scalp chloromas as the initial manifestation of infant AML.

## 1. Introduction

AML constitutes 15%–20% of pediatric leukemia cases; however, infants younger than 1 year are rarely affected, representing less than 5% of cases. These patients frequently exhibit atypical extramedullary manifestations, which can delay diagnosis [1].

The FAB M4 subtype, or acute myelomonocytic leukemia, is characterized by a prominent monocytic component, tissue infiltration, and frequent extramedullary disease [2]. Myeloid sarcomas (chloromas) occur in up to 23% of pediatric AML cases, most commonly affecting the skin, orbits, and gingiva. Testicular involvement occurs in 5%–13% of males, while scalp lesions are exceedingly rare [3].

The concurrence of bilateral testicular enlargement and scalp chloromas in an infant is exceptionally rare and presents a significant risk of misdiagnosis. Diagnosis requires a comprehensive hematologic, immunophenotypic, and imaging assessment in accordance with WHO classification criteria [2]. Current guidelines recommend intensive systemic chemotherapy as initial treatment, reserving local therapies for refractory cases [4]. These protocols achieve remission in over 85% of infants at favorable risk, obviating the need for routine stem cell transplantation [5].

This report describes a 10‐month‐old boy with bilateral testicular swelling and scalp chloromas as the initial manifestation of FAB‐M4 AML, who achieved sustained minimal residual disease (MRD)–negative remission with chemotherapy alone.

## 2. Case Presentation

A 10‐month‐old boy, born at term without perinatal complications and with no family history of chronic disease or malignancy, presented to the pediatric surgical clinic at Queen Rania Children’s Hospital in October 2024 with a 5‐day history of bilateral testicular swelling. The examination revealed nontender bilateral scrotal swelling with hydrocele. An initial complete blood count demonstrated leukocytosis (WBC 45 × 10^9^/L), prompting referral to the pediatric hematology and oncology ward.

Upon admission to the hematology ward, the patient was well‐nourished, alert, and in no distress. Vital signs were age‐appropriate: temperature 36.8°C, heart rate 110 bpm, respiratory rate 28 bpm, and blood pressure 85/55 mmHg. Weight and length were at the 50th percentile for age.

Head and neck examination revealed several small, well‐defined, nontender scalp swellings in the right and left parietal and right frontotemporal regions. These lesions were firm, mobile, and without overlying skin changes.

Abdominal examination was unremarkable, with no hepatosplenomegaly. No lymphadenopathy was detected in the cervical, axillary, or inguinal regions. Genital examination showed bilateral testicular enlargement with hydrocele; both testes were firm, nontender, mobile, and had normal overlying skin.

Chest examination was normal, with clear breath sounds and no respiratory distress. Cardiac examination revealed normal heart sounds without murmurs. Neurological assessment showed normal tone, strength, and reflexes for age, with no focal deficits.

Initial laboratory tests revealed normochromic normocytic anemia (hemoglobin 10.4 g/dL, hematocrit 33%), thrombocytosis (platelets 448 × 10^9^/L), and leukocytosis (WBC 45 × 10^9^/L) with 50% blasts on peripheral smear. Coagulation, renal, and liver function tests were normal. CRP was 17 mg/L, ESR 8 mm/hr, and LDH 390 U/L.

Scrotal ultrasound confirmed bilateral testicular enlargement (left: 3.6 × 1.4 cm; right: 3.6 × 1.8 cm) (Figure 1), with heterogeneous echotexture and increased vascularity on Doppler. Brain CT scan for scalp swellings revealed several small, well‐defined lesions in the right and left parietal and right frontotemporal regions, without bone erosion or destruction (Figure 2).

**FIGURE 1:**
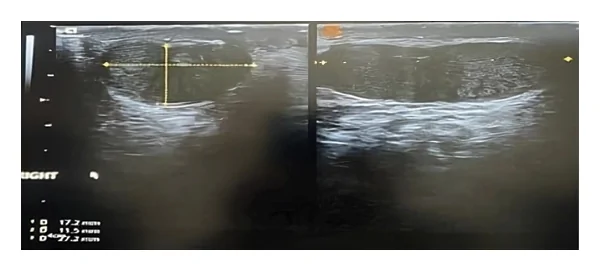
Scrotal ultrasound showing bilateral testicular masses with heterogeneous echotexture and hypervascularity.

**FIGURE 2:**
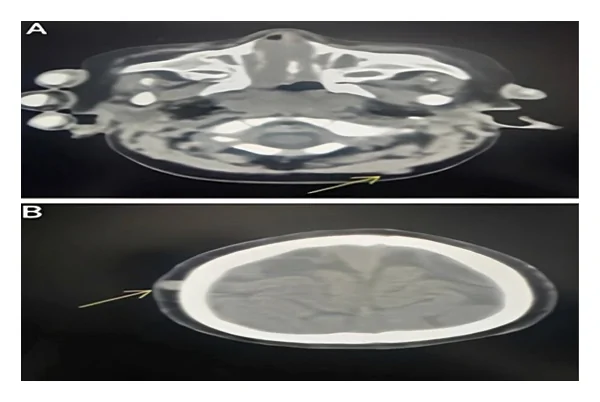
(A) Axial neck CT with submandibular mass (yellow arrow). (B) Axial brain CT showing right parietal scalp mass (yellow arrow).

Given the elevated white blood cell count and peripheral blasts, further evaluation was performed. Bone marrow aspirate and biopsy confirmed acute myelomonocytic leukemia (FAB‐M4). Immunophenotyping demonstrated blasts positive for CD13, CD33, CD34, HLA‐DR, CD64, CD4, CD14, CD11b, and myeloperoxidase (Figure 3). Cytogenetic analysis showed a normal karyotype, with negative results for 11q23/KMT2A rearrangement, Trisomy 8, Monosomy 7, inv(16), and t(8; 21). Molecular testing for FLT3‐ITD, NPM1, NRAS, KRAS, DNMT3A, TET2, and IDH1/IDH2 was not available in our laboratory.

**FIGURE 3:**
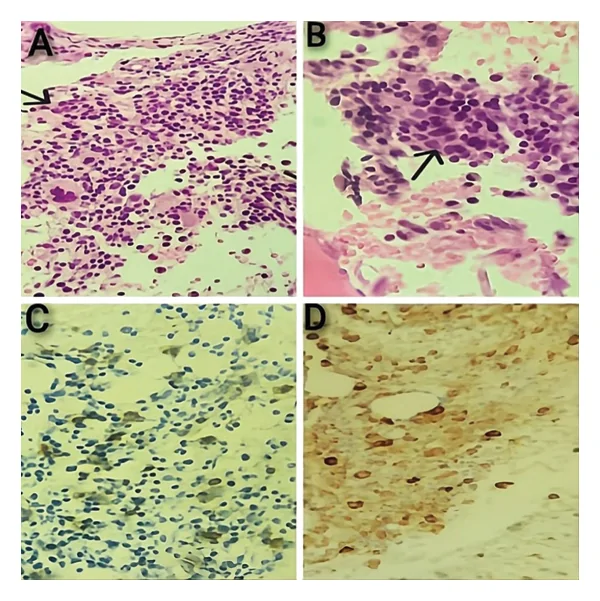
(A) MPO immunostaining showing myeloid lineage of blast cells. (B) CD34 immunostaining highlights blast cells with granular cytoplasmic staining. (C) H&E section (20*x*) showing blasts admixed with other hematopoietic elements. (D) H&E section (10*x*) showing hypercellular marrow with erythroid, myeloid precursors, and blasts.

The patient was diagnosed with AML (FAB‐M4) with testicular and scalp chloromas and initiated on an AML chemotherapy protocol.

During the initial hospitalization, serial CBCs showed a rising white blood cell count that peaked at 97 × 10^9^/L despite supportive care, indicating disease progression. Following initiation of AML chemotherapy, the WBC count normalized. Table 1 summarizes the CBC values during this period.

**TABLE 1:** Serial complete blood count (CBC) trends during initial hospitalization.

Day of admission	Hb (g/dL)	PCV (%)	MCV (fL)	Plt (× 10^9^/L)	WBC (× 10^9^/L)	ANC (× 10^9^/L)	ALC (× 10^9^/L)	Monocyte (%)	LDH (U/L)
1	10.4	33	67	448	45	7.3	23.2	10.4	390
3	9.9	29	68	450	55	11.3	28.1	14	—
5	8.5	27.5	70	367	95	22.3	36.6	35	732
6	7.8	25	68	347	86	18.5	38.5	26	—
7	7.6	25	70	293	97	16.7	32.6	44	—
9	7.3	23.1	66	179	51	31.1	14.1	23.2	540
11	8.7	25.9	68	44	6.9	3.2	2.5	1.1	—
12	8.6	26	68	29	3.7	1.5	2.1	0	348

The patient received multiagent AML chemotherapy, including cytarabine, an anthracycline, and etoposide during induction, consistent with Children’s Oncology Group (COG) treatment protocols [6]. After the first cycle, testicular swelling markedly improved, and scalp lesions resolved. Bone marrow evaluation showed that blasts had decreased to 5%–10%, but remission had not yet been achieved.

A second chemotherapy cycle was administered. Subsequent bone marrow analysis showed no blasts or evidence of acute leukemia. MRD testing confirmed complete remission.

The patient has remained in complete remission and has been followed every 2 months in our clinic since March 2025, with no evidence of relapse at the most recent visit. Follow‐up assessments included clinical evaluation, complete blood count, peripheral blood film, testicular ultrasound, and skull imaging as indicated. Table 2 summarizes the chronological course of diagnosis, chemotherapy cycles, MRD assessment, end of treatment, and follow‐up.

**TABLE 2:** Chronological timeline of treatment and disease response.

Week	Event	Clinical significance
Week 1	Diagnosis established and Cycle 1 chemotherapy started.	Initial treatment was initiated promptly after diagnosis.
Week 3	Cycle 2 chemotherapy administered.	Ongoing protocol‐based treatment continued.
Week 5	MRD was negative.	Early treatment response was favorable, with no detectable minimal residual disease.
Week 7	Cycle 3 chemotherapy given.	Consolidation treatment proceeded as planned.
Week 11	Cycle 4 chemotherapy given.	Further protocol‐based therapy completed.
Week 15	Cycle 5 chemotherapy given.	Treatment course advanced toward completion.
Week 19	MRD remained negative; end of treatment reached.	Complete response was sustained through completion of therapy.
Posttreatment follow‐up	Regular follow‐up continued.	Ongoing monitoring for relapse and late complications.

## 3. Discussion

AML with extramedullary involvement is rare in infants, and simultaneous involvement of multiple sites at diagnosis is even less common. Infant AML exhibits distinct clinical features compared to other age groups. Reports of infants presenting with both testicular and scalp disease are exceedingly rare. Although some children with AML develop extramedullary symptoms, these are less frequently observed in infants, complicating diagnosis in this population [7, 8].

In this case, initial signs of AML included bilateral testicular swelling and scalp masses, which resembled benign dermatologic or acute surgical conditions. Such presentations complicate diagnosis, as testicular involvement in childhood leukemia is uncommon at onset and more often seen at relapse [9]. This atypical presentation may delay hematologic evaluation and underscores the need for heightened clinical vigilance.

The diagnosis of FAB‐M4 AML may help explain the extensive extramedullary disease observed. Myelomonocytic and monocytic AML subtypes have a recognized association with extramedullary involvement, including myeloid sarcoma (chloroma). Clinical studies have shown that FAB‐M4 and FAB‐M5 morphology is more frequent among AML cases with extramedullary disease [10, 11].

Testicular chloromas in infancy are rarely suspected at presentation and are frequently misdiagnosed as infection, hydrocele, or torsion [12]. In this case, the absence of inflammatory signs, bilateral testicular involvement, and concurrent scalp masses prompted early consideration of malignancy and expedited hematologic evaluation. These findings indicate that atypical bilateral testicular enlargement in infants, particularly when accompanied by other extramedullary lesions, should raise suspicion for systemic malignancy [12]. Systemic chemotherapy remains the primary treatment for extramedullary AML, even when the disease involves protected anatomical sites. Local therapies such as radiotherapy or surgery are generally reserved for refractory or relapsed disease [4]. In this patient, adherence to the chemotherapy protocol alone resulted in complete resolution of both testicular and scalp disease, demonstrating that systemic treatment can be effective without local interventions when there is a favorable early response.

The patient received induction therapy with cytarabine, anthracycline, and etoposide according to group guidelines, achieving complete remission. MRD testing confirmed a robust response. MRD is now a key prognostic indicator in pediatric AML and informs treatment decisions [13]. While gemtuzumab may reduce relapse risk in select patients [14], standard regimens remain highly effective when initiated promptly and monitored closely, even in resource‐limited settings [5].

The role of hematopoietic stem cell transplantation (HSCT) in first remission for infant AML remains controversial, with decisions typically based on cytogenetic, molecular, and MRD risk stratification [15]. In this case, the patient remained MRD‐negative and lacked high‐risk genetic features, so consolidation was achieved with chemotherapy alone, avoiding HSCT‐related risks.

This case adds to the limited literature on infant AML presenting with concurrent testicular and scalp chloromas. It emphasizes the importance of early recognition of atypical presentations, multidisciplinary collaboration, and adherence to established treatment protocols to achieve favorable outcomes in these rare and complex cases.

## 4. Limitations

This report has several limitations. As a single‐case description, the findings are not generalizable. Molecular studies for recurrent AML‐associated mutations were unavailable in our laboratory, limiting comprehensive genetic characterization. Additionally, the follow‐up period is relatively short.

## 5. Conclusion

This case of infant AML with bilateral testicular and scalp chloromas demonstrates the potential for extramedullary disease to be misdiagnosed as a surgical emergency. The aggressive tissue involvement aligns with FAB‐M4 biology. Chemotherapy, administered according to established protocols, resulted in a durable MRD‐negative remission without the need for local therapies or transplantation, which is particularly significant in resource‐limited settings. Prompt referral to a hematologist for infants presenting with unusual bilateral testicular masses and close collaboration with other specialties are essential to avoid diagnostic delays. This rare case further supports the use of standardized COG‐guided treatment for favorable‐risk patients, as comparable outcomes can be achieved even in resource‐limited settings.

## Author Contributions

Ruba Alhazaimeh and Mais Jazazi prepared the initial manuscript draft and contributed to literature review. Ayman Alhwayan and Maher Khader conceived the report, supervised the clinical work, and critically revised the manuscript. Sulieman Jaafreh assisted in manuscript preparation and revision. Hazem Haddad interpreted the imaging findings and contributed to manuscript revision. Adnan Bwaneh was involved in surgical assessment and contributed to revision of the clinical description. Tasneem Malahmeh reviewed the pathology and laboratory findings and contributed to manuscript revision. Ayman Alhwayan had full access to all of the data in this study and takes complete responsibility for the integrity of the data and the accuracy of the data analysis.

## Funding

The authors received no specific funding for this work.

## Disclosure

All authors have read and approved the final version of the manuscript.

## Ethics Statement

The General Headquarters of the Jordan Armed Forces, Directorate of Royal Medical Services, and the Human Research Ethics Committee approved this study in meeting number 2/2026 on 3/3/2026.

## Consent

Written informed consent for publication of the case details and accompanying images was obtained from the patient’s legal guardian.

## Conflicts of Interest

The authors declare no conflicts of interest.

## Supporting Information

Additional supporting information can be found online in the Supporting Information section.

## Supporting information


**Supporting Information** CARE‐checklist‐English‐2013.

## Data Availability

The data supporting the findings of this case report are available from the corresponding author upon reasonable request, subject to patient privacy and institutional policy.

## References

[1] De R. , Jasmijn D. E. , Michel Zwaan C. , and Van den Heuvel-Eibrink M. , Pediatric AML: From Biology to Clinical Management, Journal of Clinical Medicine. (2015) 4, no. 1, 127–149, 10.3390/jcm4010127. PMC4470244 26237023

[2] Arber D. A. , Orazi A. , Hasserjian R. et al., The 2016 Revision to the World Health Organization Classification of Myeloid Neoplasms and Acute Leukemia, Blood, The Journal of the American Society of Hematology. (2016) 127, no. 20, 2391–2405, 10.1182/blood-2016-03-643544. 27069254

[3] Li W. , Shi M. , Zhou P. et al., Extramedullary Infiltration in Pediatric Acute Myeloid Leukemia: Results From the Therapeutically Applicable Research to Generate Effective Treatments (TARGET) Initiative, Pediatric Blood and Cancer. (2024) 71, no. 7, 10.1002/pbc.31014. 38644612

[4] Bakst R. L. , Tallman M. S. , Douer D. , and Joachim Y. , How I Treat Extramedullary Acute Myeloid Leukemia, Blood, The Journal of the American Society of Hematology. (2011) 118, no. 14, 3785–3793, 10.1182/blood-2011-04-347229. 21795742

[5] Gamis A. S. , Alonzo T. A. , Perentesis J. P. , and Meshinchi S. , COG Acute Myeloid Leukemia Committee. Children’s Oncology Group’s 2013 Blueprint for Research: Acute Myeloid Leukemia, Pediatric Blood and Cancer. (2013) 60, no. 6, 964–971, 10.1002/pbc.24432. PMC4605815 23255301

[6] Arnaout M. K. , Radomski K. M. , Srivastava D. K. et al., Treatment of Childhood Acute Myelogenous Leukemia With an Intensive Regimen (AML-87) That Individualizes Etoposide and Cytarabine Dosages: Short-and Long-Term Effects, Leukemia. (2000) 14, no. 10, 1736–1742, 10.1038/sj.leu.2401906. 11021748

[7] Masetti R. , Vendemini F. , Zama D. , Biagi C. , Pession A. , and Locatelli F. , Acute Myeloid Leukemia in Infants: Biology and Treatment, Frontiers in Pediatrics. (2015) 3, 10.3389/fped.2015.00037. PMC4411976 25973412

[8] Zwaan C. M. , Kolb E. A. , Reinhardt D. et al., Collaborative Efforts Driving Progress in Pediatric Acute Myeloid Leukemia, Journal of Clinical Oncology. (2015) 33, no. 27, 2949–2962, 10.1200/JCO.2015.62.8289. PMC4567700 26304895

[9] Byrd J. C. , Edenfield W. J. , Shields D. J. , and Dawson N. A. , Extramedullary Myeloid Cell Tumors in Acute Nonlymphocytic Leukemia: A Clinical Review, Journal of Clinical Oncology: Official Journal of the American Society of Clinical Oncology. (1995) 13, no. 7, 1800–1816, 10.1200/JCO.1995.13.7.1800. 7602369

[10] Pileri S. A. , Ascani S. , Cox M. C. et al., Myeloid Sarcoma: Clinico-Pathologic, Phenotypic and Cytogenetic Analysis of 92 Adult Patients, Leukemia. (2007) 21, no. 2, 340–350, 10.1038/sj.leu.2404491. 17170724

[11] Støve H. K. , Damgaard Sandahl J. , Abrahamsson J. et al., Extramedullary Leukemia in Children With Acute Myeloid Leukemia: A Population‐Based Cohort Study From the Nordic Society of Pediatric Hematology and Oncology (NOPHO), Pediatric Blood and Cancer. (2017) 64, no. 12, 10.1002/pbc.26520. 28333413

[12] Sahu K. K. , Adrian Sherif A. , Mishra A. K. , Lal A. , and Singh A. , Testicular Myeloid Sarcoma: A Systematic Review of the Literature, Clinical Lymphoma, Myeloma & Leukemia. (2019) 19, no. 10, 603–618, 10.1016/j.clml.2019.04.013. 31371220

[13] Karol S. E. , Coustan-Smith E. , Pounds S. et al., Clinical Impact of Minimal Residual Disease in Blood and Bone Marrow of Children With Acute Myeloid Leukemia, Blood Advances. (2023) 7, no. 14, 3651–3657, 10.1182/bloodadvances.2022009534. PMC10365945 37058475

[14] Gamis A. S. , Alonzo T. A. , Meshinchi S. et al., Gemtuzumab Ozogamicin in Children and Adolescents With de Novo Acute Myeloid Leukemia Improves Event-Free Survival by Reducing Relapse Risk: Results From the Randomized Phase III Children’s Oncology Group Trial AAML0531, Journal of Clinical Oncology: Official Journal of the American Society of Clinical Oncology. (2014) 32, no. 27, 3021–3032, 10.1200/JCO.2014.55.3628. PMC4162498 25092781

[15] Masetti R. , Muratore E. , Gori D. , Prete A. , and Franco L. , Allogeneic Hematopoietic Stem Cell Transplantation for Pediatric Acute Myeloid Leukemia in First Complete Remission: A Meta-Analysis, Annals of Hematology. (2022) 101, no. 11, 2497–2506, 10.1007/s00277-022-04965-x. PMC9546991 36038660

