# Supplementary material for: Atypical Extramedullary Acute Myeloid Leukemia in Infancy: Bilateral Testicular Swelling and Scalp Lesions at Diagnosis
Source: Case Rep Hematol. 2026 Oct 12;2026:2024335. doi: 10.1155/crh/2024335 (PMC13659258; doi:10.1155/crh/2024335)
Supplement: Supplementary file 1 [file CRH-2026-2024335-s001.pdf]

CARE Checklist of information to include when writing a case report

Topic	Item	Checklist item description	Reported on Line
**Title**	**1**	The diagnosis or intervention of primary focus followed by the words "case report"	Atypical Extramedullary Acute Myeloid Leukemia in Infancy: Bilateral Testicular Swelling and Scalp Lesions at Diagnosis
**Key Words**	**2**	2 to 5 key words that identify diagnoses or interventions in this case report, including "case report"	Acute Myeloid Leukemia, Myeloid Sarcoma, Testicular Neoplasms, Acute Myelomonocytic Leukemia, Case Report
**Abstract (no references)**	**3a**	Introduction: What is unique about this case and what does it add to the scientific literature?	This case highlights the importance of early hematologic evaluation in infants with bilateral testicular masses to avoid misdiagnosis and illustrates the rare occurrence of concurrent testicular and scalp chloromas as the initial manifestation of infant AML
	**3b**	Main symptoms and/or important clinical findings	We report a 10-month-old male infant who presented with firm bilateral testicular enlargement and multiple parietal scalp nodules, accompanied by leukocytosis and circulating blasts in peripheral blood
	**3c**	The main diagnoses, therapeutic interventions, and outcomes	Bone marrow examination confirmed French-American-British (FAB) M4 AML. Scrotal ultrasound demonstrated heterogeneous masses, and computed tomography revealed scalp chloromas without bone erosion. The patient was treated with AML chemotherapy, which resulted in resolution of the extramedullary lesions and achievement of remission
	**3d**	Conclusion—What is the main "take-away" lesson(s) from this case?	This case highlights the importance of early hematologic evaluation in infants with bilateral testicular masses to avoid misdiagnosis and illustrates the rare occurrence of concurrent testicular and scalp chloromas as the initial manifestation of infant AML
**Introduction**	**4**	One or two paragraphs summarizing why this case is unique (may include references)	Acute myeloid leukemia (AML) constitutes 15–20% of pediatric leukemia cases; however, infants younger than one year are rarely affected, representing less than 5% of cases.<br>The concurrence of bilateral testicular enlargement and scalp chloromas in an infant is exceptionally rare and presents a significant risk of misdiagnosis.
**Patient Information**	**5a**	De-identified patient specific information.	A 10-month-old boy, born full term without complications and with no family history of chronic disease or cancer
	**5b**	Primary concerns and symptoms of the patient.	presented to the pediatric surgical clinic with five days of bilateral testicular swelling. Examination revealed non-tender swelling and a hydrocele
	**5c**	Medical, family, and psycho-social history including relevant genetic information	with no family history of chronic disease or cancer

	**5d**	Relevant past interventions with outcomes	N/A
**Clinical Findings**	**6**	Describe significant physical examination (PE) and important clinical findings.	Upon admission to the hematology ward, the patient was well-nourished, alert, and in no distress. Head and neck examination revealed several small, well-defined, non-tender scalp swellings... Genital examination showed bilateral testicular enlargement with hydrocele... Chest examination was normal... Cardiac examination revealed normal heart sounds..
**Timeline**	**7**	Historical and current information from this episode of care organized as a timeline	Table 2
**Diagnostic Assessment**	**8a**	Diagnostic testing (such as PE, laboratory testing, imaging, surveys).	normochromic normocytic anemia (hemoglobin 10.4 g/dL, hematocrit 33%), thrombocytosis (platelets 448×10^9/L), and leukocytosis (WBC 45×10^9/L) with 50% blasts on peripheral smear... CRP was 17 mg/L, ESR 8 mm/hr, and LDH 390 U/L," along with serial CBC trends in Table 1. Imaging includes: "Scrotal ultrasound confirmed bilateral testicular enlargement...
	**8b**	Diagnostic challenges (such as access to testing, financial, or cultural)	Molecular testing for FLT3-ITD, NPM1, NRAS, KRAS, DNMT3A, TET2, and IDH1/IDH2 was not available in our laboratory
	**8c**	Diagnosis (including other diagnoses considered)	Bone marrow aspirate and biopsy confirmed acute myelomonocytic leukemia (FAB-M4)" and then, "The patient was diagnosed with AML (FAB-M4) with testicular and scalp chloromas
	**8d**	Prognosis (such as staging in oncology) where applicable	Cytogenetic analysis showed a normal karyotype, with negative results for 11q23/KMT2A rearrangement, trisomy 8, monosomy 7, inv16, and t(8;21) and Minimal residual disease testing confirmed complete remission
**Therapeutic Intervention**	**9a**	Types of therapeutic intervention (such as pharmacologic, surgical, preventive, self-care)	Multiagent systemic chemotherapy (cytarabine + anthracycline ± etoposide) per COG protocol
	**9b**	Administration of therapeutic intervention (such as dosage, strength, duration)	5 cycles total, exact dosages not specified in manuscript.
	**9c**	Changes in therapeutic intervention (with rationale)	NA
**Follow-up and Outcomes**	**10a**	Clinician and patient-assessed outcomes (if available)	Minimal residual disease testing confirmed complete remission
	**10b**	Important follow-up diagnostic and other test results	Bone marrow cleared of blasts, MRD negative, and follow-up CBC/imaging used for surveillance
	**10c**	Intervention adherence and tolerability (How was this assessed?)	not specifically reported
	**10d**	Adverse and unanticipated events	NA
**Discussion**	**11a**	A scientific discussion of the strengths AND limitations associated with this case report	This case contributes to the limited reports of infant AML presenting with concurrent testicular and scalp chloromas. It underscores the importance of early recognition of atypical presentations, multidisciplinary collaboration, and adherence to treatment protocols…". The

			limitations are stated in the separate Limitations section.
	**11b**	Discussion of the relevant medical literature with references.	The key literature-backed discussion appears in the paragraphs starting from “Acute myeloid leukemia (AML) with extramedullary involvement is rare in infants...” through the section discussing MRD and HSCT
	**11c**	The scientific rationale for any conclusions (including assessment of possible causes)	The diagnosis of FAB-M4 AML may help explain the extensive extramedullary disease observed. Myelomonocytic and monocytic AML subtypes have a recognized association with extramedullary involvement..
	**11d**	The primary “take-away” lessons of this case report (without references) in a one paragraph conclusion	The final paragraph states the main lessons clearly: the case shows that infant AML with bilateral testicular and scalp chloromas can mimic a surgical emergency, that chemotherapy can achieve durable MRD-negative remission, and that early hematology referral plus multidisciplinary care helps avoid diagnostic delay
**Patient Perspective**	**12**	The patient should share their perspective in one to two paragraphs on the treatment(s) they received	NA
**Informed Consent**	**13**	Did the patient give informed consent? Please provide if requested	Yes No
